# Correction: Zbtb16 regulates social cognitive behaviors and neocortical development

**DOI:** 10.1038/s41398-021-01402-x

**Published:** 2021-05-11

**Authors:** Noriyoshi Usui, Stefano Berto, Ami Konishi, Makoto Kondo, Genevieve Konopka, Hideo Matsuzaki, Shoichi Shimada

**Affiliations:** 1 Department of Neuroscience and Cell Biology, Graduate School of Medicine, Osaka University, Osaka, 565-0871 Japan; 2 Department of Child Development, United Graduate School of Child Development, Osaka University, Osaka, 565-0871 Japan; 3 Global Center for Medical Engineering and Informatics, Osaka University, Osaka, 565-0871 Japan; 4 Addiction Research Unit, Osaka Psychiatric Research Center, Osaka Psychiatric Medical Center, Osaka, 541-8567 Japan; 5 Department of Neuroscience, University of Texas Southwestern Medical Center, Dallas, TX 75390 USA; 6 Division of Development of Mental Functions, Research Center for Child Mental Development, University of Fukui, Fukui, 910-1193 Japan; 7 Life Science Innovation Center, University of Fukui, Fukui, 910-1193 Japan

**Keywords:** Molecular neuroscience, Autism spectrum disorders, Schizophrenia

Correction to: *Translational Psychiatry*

10.1038/s41398-021-01358-y published online 24 April 2021

The original version of this article unfortunately contained a mistake in the following sentence “These analyses indicate that the Ztbt16-regulated transcriptome is related to both ASD and SCZ”. “Ztbt16” should be changed to “Zbtb16”. We apologize for the error. The original article has been corrected.

