# Supplementary material for: Zbtb16 regulates social cognitive behaviors and neocortical development
Source: Transl Psychiatry. 2021 Apr 24;11:242. doi: 10.1038/s41398-021-01358-y (PMC8068730; doi:10.1038/s41398-021-01358-y)
Supplement: Supplementary file 1 — Supplementary information [file 41398_2021_1358_MOESM1_ESM.docx]

Supplementary information

***Zbtb16* regulates social cognitive behaviors and neocortical development.**

Noriyoshi Usui, Stefano Berto, Ami Konishi, Makoto Kondo, Genevieve Konopka, Hideo Matsuzaki, and Shoichi Shimada


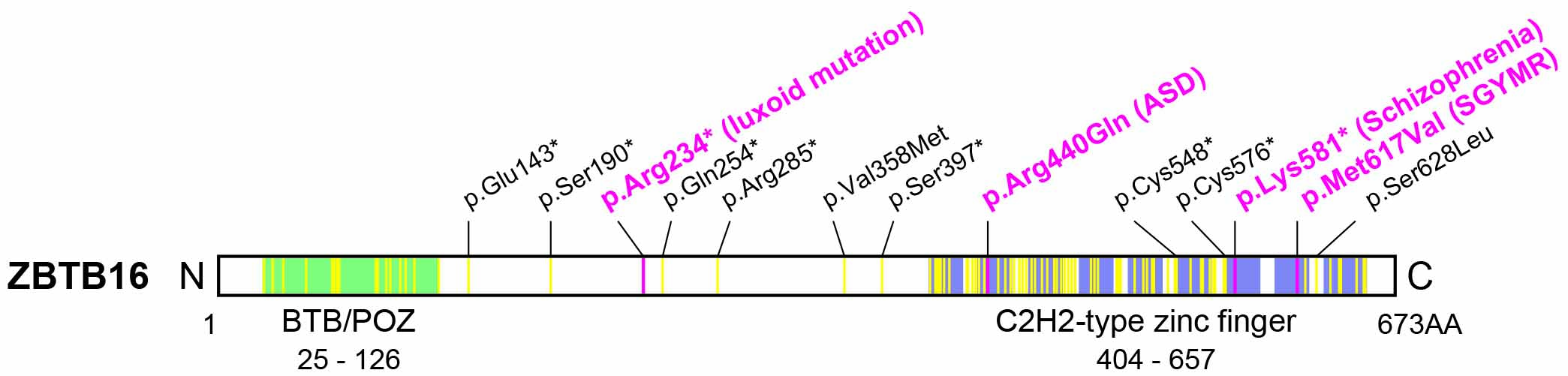


**Supplementary Figure 1. *Zbtb16* mutations and protein domains.**

Schematic of ZBTB16 protein showing the location of human mutations. The *ZBTB16* gene is located in 11q23.2. Duplications of 11q13.2-q25 have been identifie­d in children who have developmental delay with or without congenital malformations^1^. Other duplications of 11q22.1-q25 and 11q23.2-q23.3 have also been identified in ASD, and intellectual and developmental disabilities^2^. A single nucleotide polymorphism (SNP) within the *ZBTB16* gene (c.1453+15115C>T; c.1454-7218C>T) showed association in the secondary analyses in a combined The Autism Genome Project (AGP) GWAS samples^3^. A spontaneous *luxoid* (*lu*) mutation (p.Arg234*) was identified in the *Zbtb16^lu^* mutant mouse^4, 5^, which is highly conserved across humans to zebrafish. A SNV (c.1849A>G [p.Met617Val]) in the C2H2-type zinc finger (ZF) domain is a causative mutation for Skeletal defects, genital hypoplasia, and mental retardation (SGYMR)^6^. A SNV (c.1319G>A [p.Arg440Gln]) in the ZF domain was identified in brothers with ASD^7^. A SNV (c.1741A>T [p.Lys581*]) in the ZF domain was identified in a patient with schizophrenia^8, 9^. Other missense and nonsense mutations (highlighted in orange) in *ZBTB16* have been reported in ClinVar^10^ (https://www.ncbi.nlm.nih.gov/clinvar) and the Human Phenotype Ontology^11^ (https://hpo.jax.org) databases. Disorder-associated mutations are highlighted in magenta. *BTB/POZ: BTB/ POZ domain for protein-protein interaction, C2H2-type zinc finger: Cys2-His2-type zinc finger domain for DNA binding, N: N-terminal, C: C-terminal, AA: amino acids.*


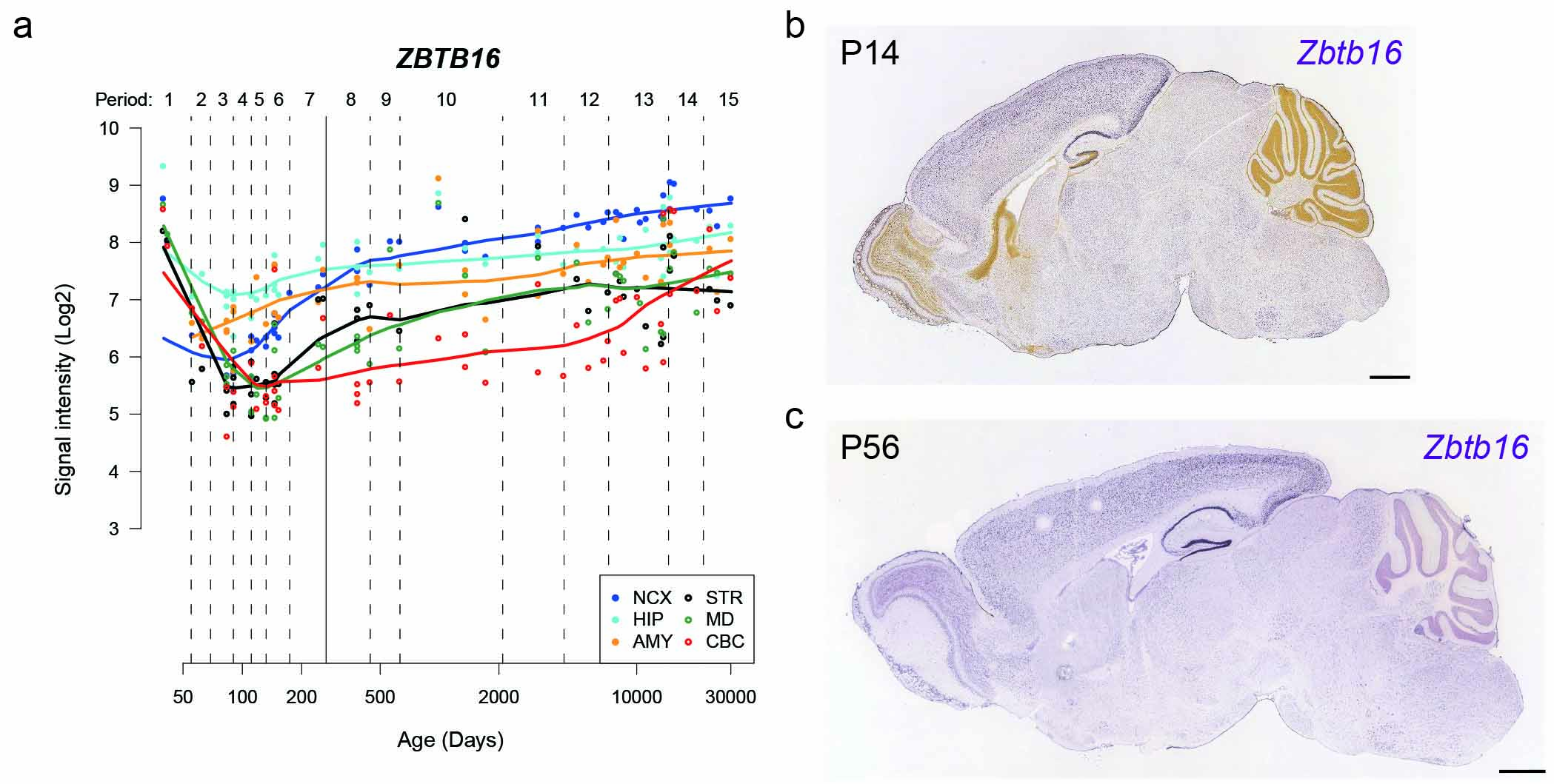


**Supplementary Figure 2. *Zbtb16* expression in the cortex.**

**a** *ZBTB16* expression trajectory in the neocortex, striatum, amygdala, hippocampus, midbrain, cerebellum from Human Brain Transcriptome (https://hbatlas.org; Kang et al. 2011). *NCX: neocortex, STR: striatum, AMG: amygdala, HIP: hippocampus, MD: midbrain, CBC: cerebellum.* **b, c** *Zbtb16* mRNA expression in sagittal sections of mouse brains at P14 (**b**) and P56 (**c**) detected by *in situ* hybridization. All images are from the Allen Brain Atlas. Scale bars: 1000 μm.


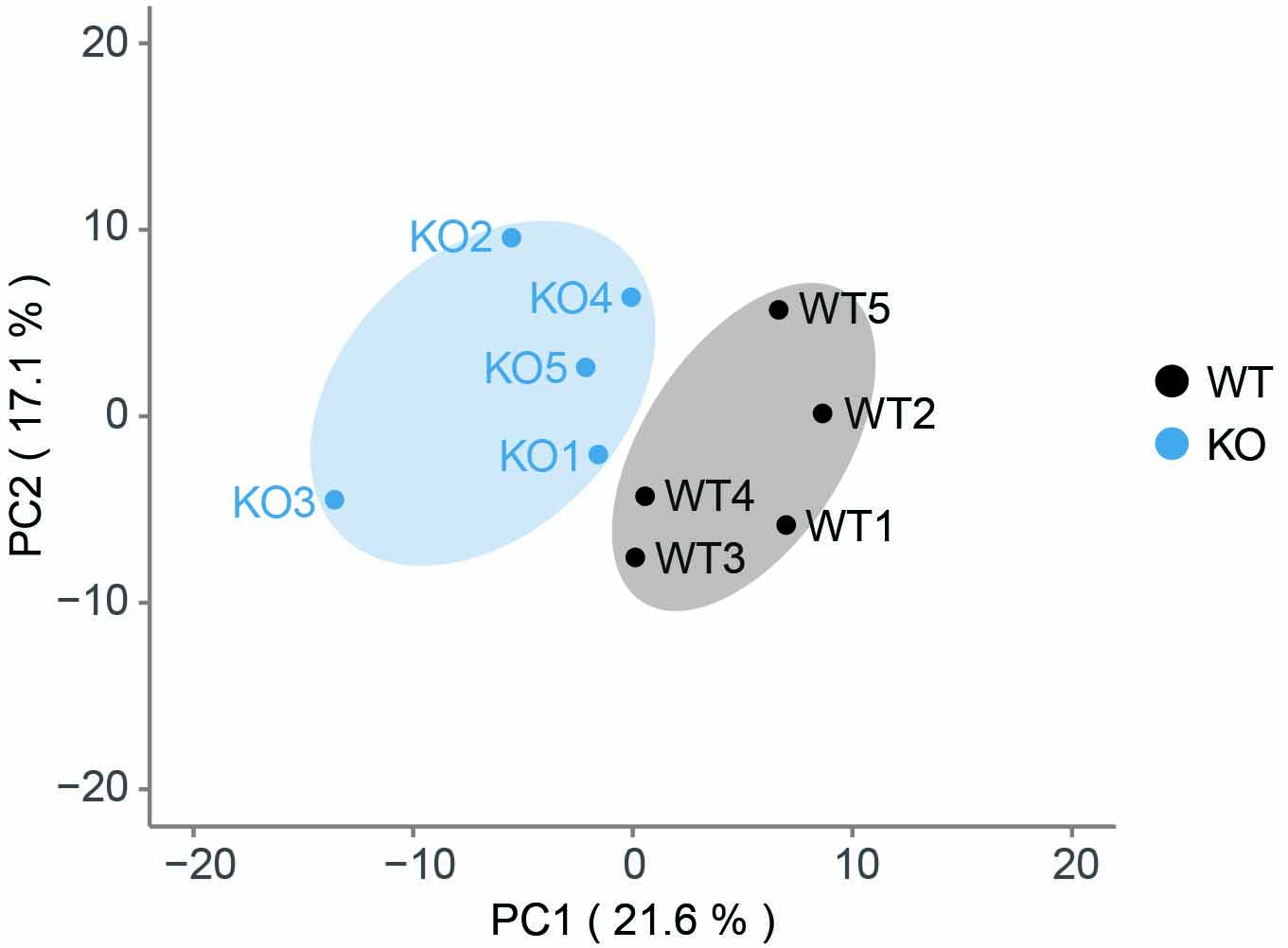


**Supplementary Figure 3. Principal component analysis (PCA) of *Zbtb16* genotypes.**

PCA in mouse prefrontal cortex, showing clear separation between WT and KO. *WT: wild-type, KO: Zbtb16 knockout.*


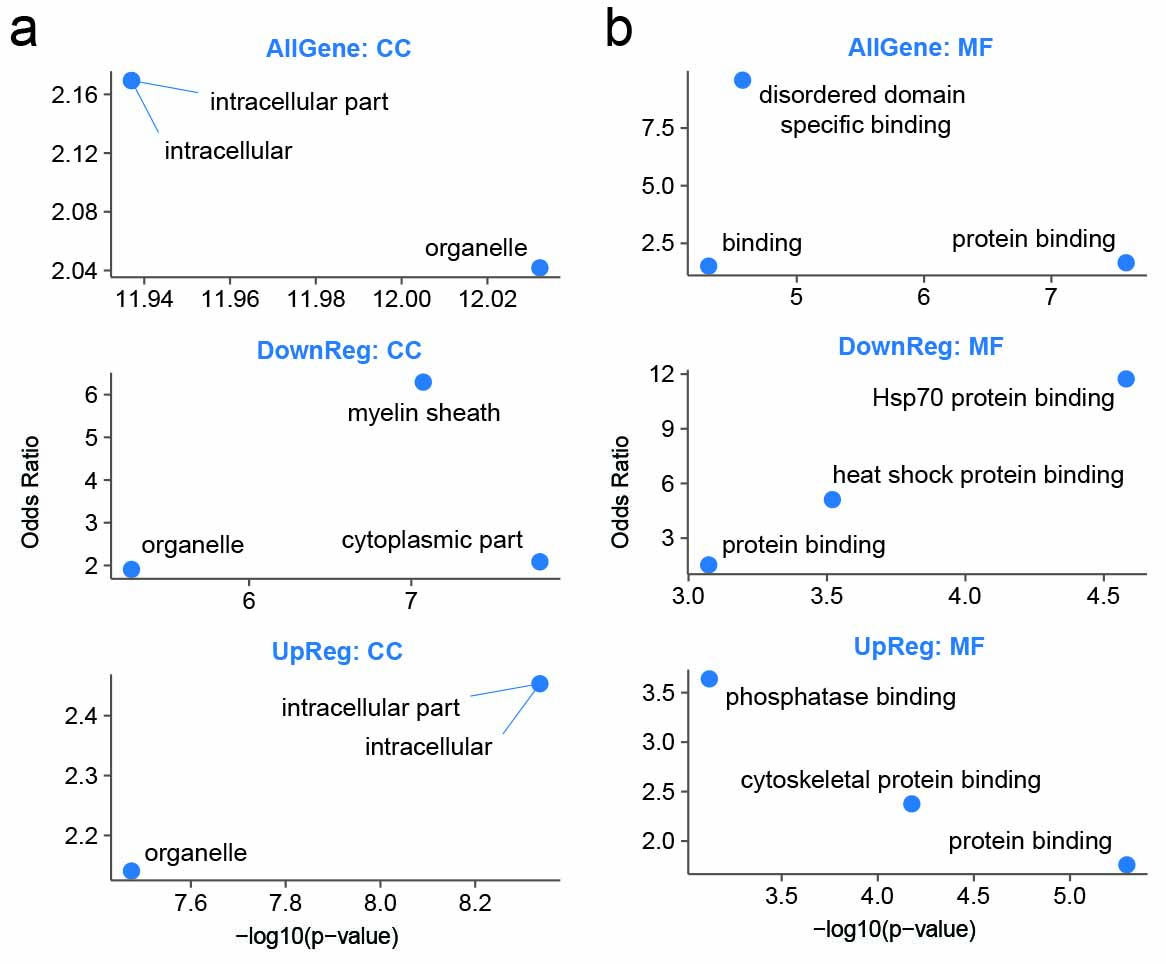


**Supplementary Figure 4. Gene ontology (GO) of *Zbtb16* differentially expressed genes (DEGs).**

**a, b** GO analyses of *Zbtb16* DEGs in cellular component (CC) (**a**), and molecular function (MF) (**b**). Scatterplots represent the top 3 functions in each module. Y-axis=Odds Ratio, X-axis=-log10(p-value). *AllGene: all DEGs, DownReg: downregulated DEGs, UpReg: upregulated DEGs.*


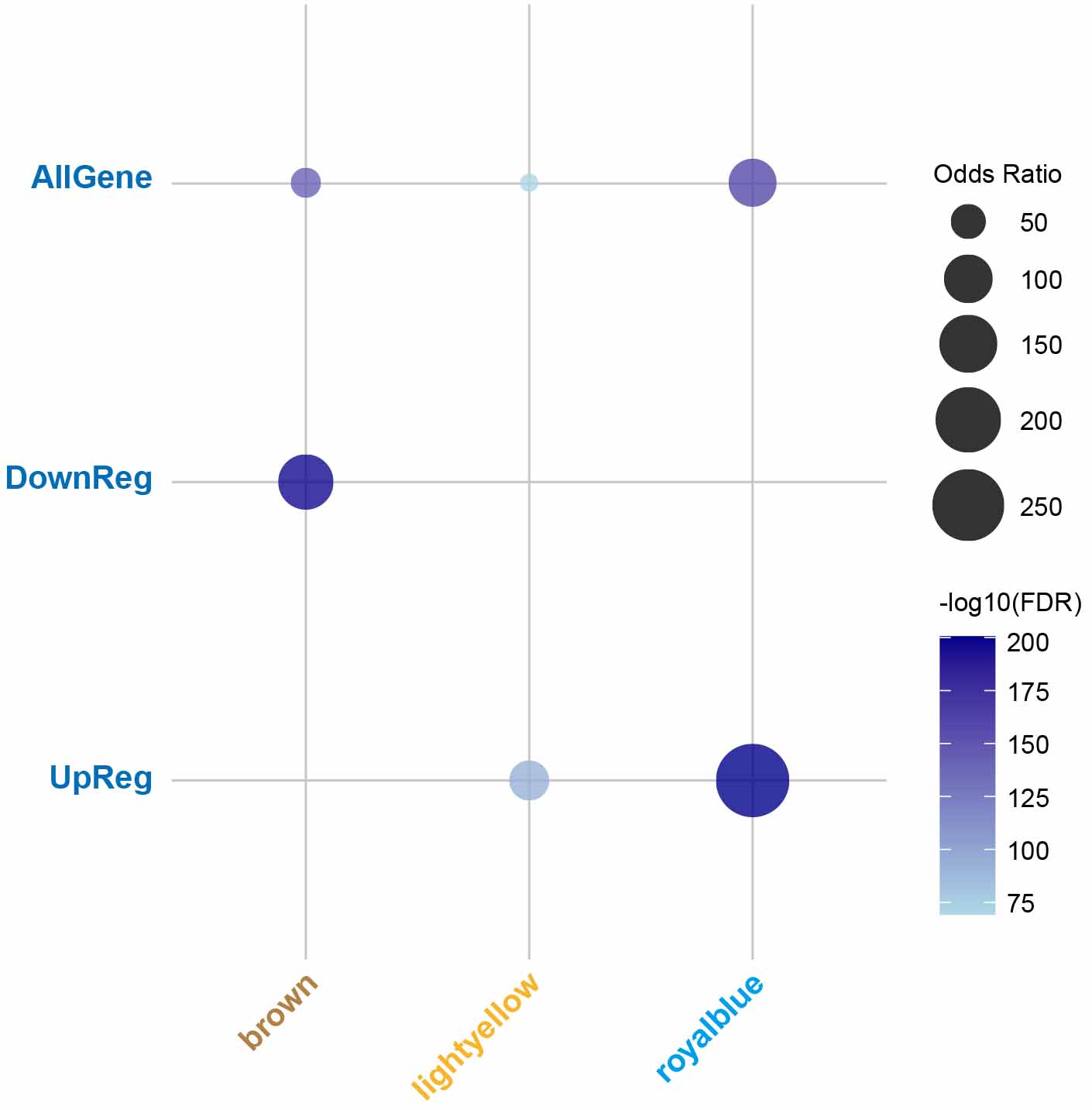


**Supplementary Figure 5. *Zbtb16* DEGs enrichments for *Zbtb16*-specific modules.**

Downregulated *Zbtb16* DEGs are enriched in brown module. Upregulated *Zbtb16* DEGs are enriched in lightyellow and royalblue modules. *AllGene: all DEGs, DownReg: downregulated DEGs, UpReg: upregulated DEGs.*


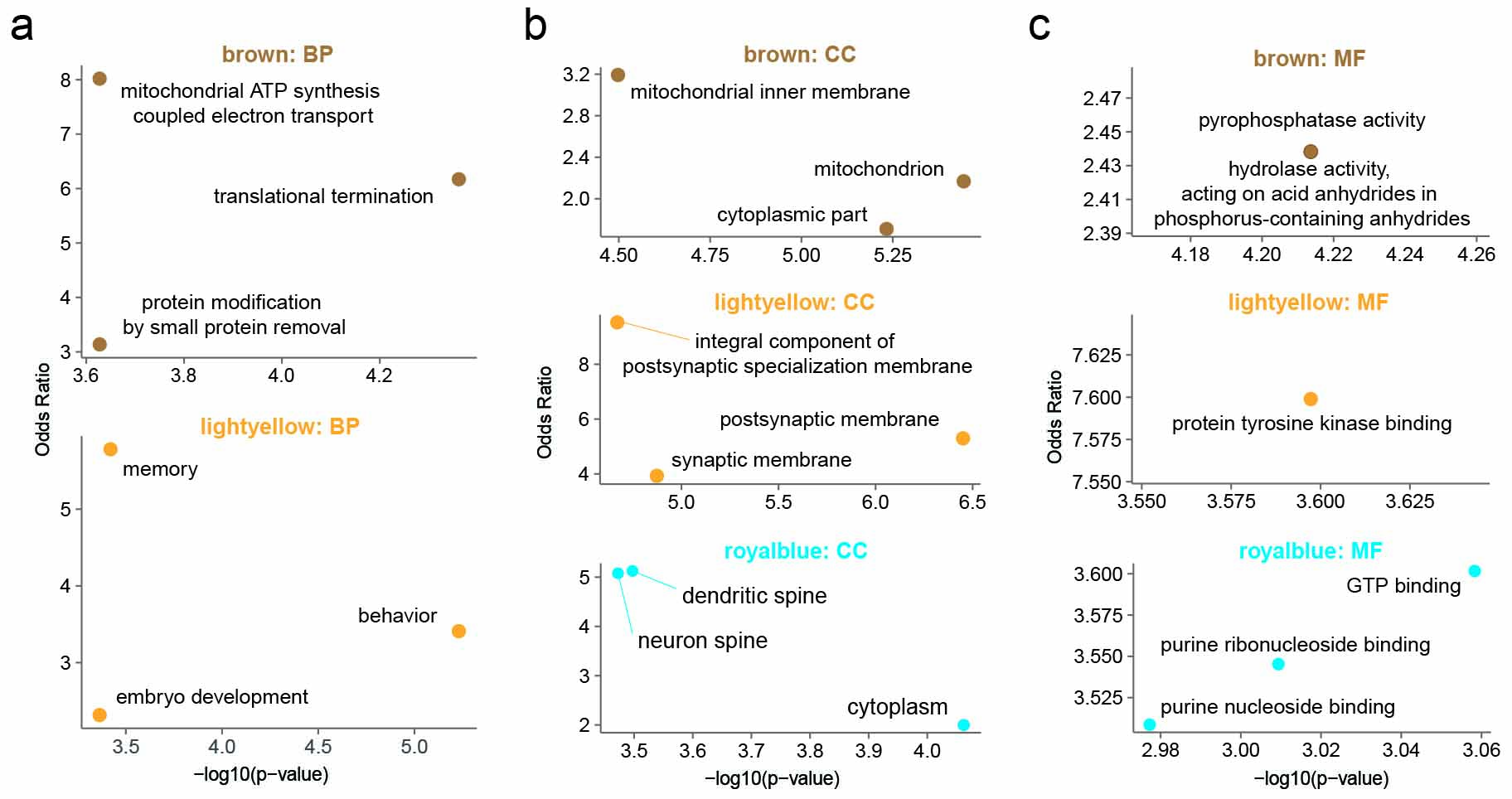


**Supplementary Figure 6. GO of *Zbtb16*-specific modules.**

**a-c** GO analyses of modules in biological process (BP) (**a**), CC (**b**), and MF (**c**). Scatterplots represent the top 3 functions in each module. Y-axis=Odds Ratio, X-axis=-log10(FDR).

**Supplementary References**

1. Girirajan S, Rosenfeld JA, Coe BP, Parikh S, Friedman N, Goldstein A *et al.* Phenotypic heterogeneity of genomic disorders and rare copy-number variants. *The New England journal of medicine* 2012; **367**(14)**:** 1321-1331.

2. Kaminsky EB, Kaul V, Paschall J, Church DM, Bunke B, Kunig D *et al.* An evidence-based approach to establish the functional and clinical significance of copy number variants in intellectual and developmental disabilities. *Genetics in medicine : official journal of the American College of Medical Genetics* 2011; **13**(9)**:** 777-784.

3. Anney R, Klei L, Pinto D, Almeida J, Bacchelli E, Baird G *et al.* Individual common variants exert weak effects on the risk for autism spectrum disorders. *Human molecular genetics* 2012; **21**(21)**:** 4781-4792.

4. Green MC. Luxoid, a new hereditary leg and foot abnormality in the house mouse. *Journal of Heredity* 1955; **46**(3)**:** 91-99.

5. Buaas FW, Kirsh AL, Sharma M, McLean DJ, Morris JL, Griswold MD *et al.* Plzf is required in adult male germ cells for stem cell self-renewal. *Nature genetics* 2004; **36**(6)**:** 647-652.

6. Fischer S, Kohlhase J, Böhm D, Schweiger B, Hoffmann D, Heitmann M *et al.* Biallelic loss of function of the promyelocytic leukaemia zinc finger (PLZF) gene causes severe skeletal defects and genital hypoplasia. *Journal of medical genetics* 2008; **45**(11)**:** 731-737.

7. Bacchelli E, Loi E, Cameli C, Moi L, Vega-Benedetti AF, Blois S *et al.* Analysis of a Sardinian Multiplex Family with Autism Spectrum Disorder Points to Post-Synaptic Density Gene Variants and Identifies CAPG as a Functionally Relevant Candidate Gene. *J Clin Med* 2019; **8**(2).

8. Purcell SM, Moran JL, Fromer M, Ruderfer D, Solovieff N, Roussos P *et al.* A polygenic burden of rare disruptive mutations in schizophrenia. *Nature* 2014; **506**(7487)**:** 185-190.

9. Fromer M, Pocklington AJ, Kavanagh DH, Williams HJ, Dwyer S, Gormley P *et al.* De novo mutations in schizophrenia implicate synaptic networks. *Nature* 2014; **506**(7487)**:** 179-184.

10. Landrum MJ, Lee JM, Riley GR, Jang W, Rubinstein WS, Church DM *et al.* ClinVar: public archive of relationships among sequence variation and human phenotype. *Nucleic acids research* 2014; **42**(Database issue)**:** D980-985.

11. Köhler S, Vasilevsky NA, Engelstad M, Foster E, McMurry J, Aymé S *et al.* The Human Phenotype Ontology in 2017. *Nucleic acids research* 2017; **45**(D1)**:** D865-d876.
